# 934. Burn Diagram Standardization: A Quality Improvement Initiative in a Regional Burn Center

**DOI:** 10.1093/jbcr/irag033.501

**Published:** 2026-04-06

**Authors:** Michaela M Craig, Sendra Starnes, Michelle McKenney, Karen Parker, Cynthia J Mastropieri, Christopher K Craig, Anju Saraswat, John Kevin Bailey, James H Holmes, IV

**Affiliations:** Atrium Health Wake Forest Baptist Burn Center, Winston-Salem, NC; Atrium Health Wake Forest Baptist, Winston-Salem, NC; Atrium Health Wake Forest Baptist, Winston-Salem, NC; Atrium Health Wake Forest Baptist, Winston-Salem, NC; Atrium Health Wake Forest Baptist, Winston-Salem, NC; Atrium Health Wake Forest Baptist Hospital / High Point University, High Point, NC; Atrium Health Wake Forest Baptist, Winston-Salem, NC; Wake Forest University School of Medicine, Winston-Salem, NC; Atrium Health Wake Forest Baptist Burn Center, Winston-Salem, NC

## Abstract

**Introduction:**

Accurate assessment of burn size and depth is critical for guiding resuscitation, monitoring patient progress, and ensuring consistency across providers. The Burn Care Quality Program (BCQP) requires both an initial and final (ultimate) assessment of burn depth and total body surface area (TBSA) for all admitted burn patients. These are typically documented using the Lund and Browder diagram on day 1 (admission) and day 3. Despite this requirement, there are no standardized recommendations regarding which team member should complete the diagram or institutional processes to ensure compliance. This quality improvement initiative aimed to evaluate and optimize compliance with burn diagram completion within a single burn center.

**Methods:**

Historically, burn bedside registered nurses completed the Lund and Browder diagram at admission and on day 3, with training delivered informally by preceptors during orientation. Beginning in March 2022, education on burn depth assessment, accurate TBSA calculation, the Fitzpatrick skin typing scale, and standardized burn photography was formally incorporated into “Burn Academy,” a structured course required for all new burn nurses within their first year of hire.

Additional process changes were introduced in 2023. In May, performance improvement nurses began sending immediate email notifications to unit leadership if either the day 1 or day 3 diagram was missing, prompting completion within 24 hours. In November 2023, the electronic diagram was modified to include an “ED Admission Date” field, addressing recurrent errors in calculating the day 3 timeline from inpatient admission rather than ED arrival.

**Results:**

Between July 2019 and February 2022, 34 burn diagrams were missing (day 1 or day 3) out of 634 adult admissions. Following implementation of Burn Academy education (March 2022–April 2024), missing diagrams decreased to 14 of 481 admissions. After introducing standardized PI-to-leadership communication (May–November 2023), only 3 of 97 admissions were missing diagrams. Since the diagram modification in December 2024, there have been zero missing diagrams across 155 consecutive admissions through July 2025.

**Conclusions:**

Stepwise implementation of structured education, real-time communication protocols, and EMR tool optimization resulted in a dramatic and sustained improvement in burn diagram compliance, culminating in 100% completion across the most recent evaluation period.

**Applicability of Research to Practice:**

This QI project demonstrates the value of targeted interventions that combine education, accountability, and system redesign. Standardizing burn diagram documentation processes improved compliance, enhanced the accuracy of patient records, and reinforced the reliability of data used for clinical decision-making and quality reporting. Similar strategies can be adapted by other burn centers to improve adherence to best practices and strengthen institutional outcomes.

**Funding for the study:**

N/A.

